# Position within the hospital and role in the emergency department of emergency physicians in the Netherlands: a national survey

**DOI:** 10.1186/s12245-020-0267-2

**Published:** 2020-02-10

**Authors:** Sarah Coppes, Rebekka Veugelers, Roger A. P. A. Hessels, Crispijn L. van den Brand, Menno I. Gaakeer

**Affiliations:** 1 Spaarne Gasthuis, Haarlem, The Netherlands; 2 ADRZ, Goes, The Netherlands; 3 ETZ, Tilburg, The Netherlands; 4 HMC, The Hague, The Netherlands

**Keywords:** Emergency medicine, Netherlands, Surveys and questionnaires, Delivery of health care

## Abstract

**Objectives:**

Emergency medicine (EM) in the Netherlands has developed rapidly and initially without central guidance. This has led to heterogeneity in current EM practice. Our aim was to quantify this heterogeneity by answering the following questions: (1) What is the current position of emergency physicians (EPs) within hospital organizations? (2) Which roles and responsibilities do EPs have across emergency departments (EDs)?

**Methods:**

During 2018, we conducted a survey among all EM consultant bodies (CBs, *n* = 56) in the Netherlands. Data was analyzed using descriptive statistics.

**Results:**

The response rate was 91.1%. Presence of EPs has been realized 24/7 in 23.1% of EDs. EPs were the main consultants for all ED patients in 9.8% of CBs, but never had this role in 13.7% of CBs. EPs supervised EM junior doctors in 78.5% of EDs, GPs in training in 80.0% of EDs, and junior doctors of other specialties in 41.5% of EDs. Procedures such as lumbar puncture (LP), procedural sedation and analgesia (PSA), and emergency ultrasound (US) were performed by all EPs in the CB in a range between 5.9 and 78.4%. In 36.9% of EDs, EPs did not analyze patients with presumed cardiac pathology due to a separate *First Heart Aid*.

**Conclusion:**

We conclude that there is a high degree of heterogeneity between emergency CBs in regard to the position in the hospital and the role or responsibilities in the ED. Lack of uniformity might inhibit emancipation of the profession.

## Introduction

In the Netherlands, emergency medicine (EM) as an independent profession started 20 years ago with the foundation of the Dutch Society of Emergency Physicians (DSEP). Before 1999, inexperienced junior doctors staffed most emergency departments (EDs) despite the growing complexity of delivered care. Different stakeholders realized that reorganization of these departments, including training of emergency physicians (EPs), could improve emergency care quality. Consequently, in 2000, the first EM residency programs in the Netherlands started [1]. The number of residency programs grew quickly; however, lack of central guidance or authority resulted in marked differences between residencies. These differences led to dissimilarities in knowledge, skills, and responsibilities between EPs [2, 3]. Besides this, local hospital needs dictated which patient categories were analyzed by EPs, thus further increasing the differences between EPs [4]. To create more uniformity in EM, a standardized nationwide 3-year residency program was developed and approved in 2008, after which the Medical Specialist Registration Committee recognized EM as an area of special interest. In the years thereafter, the DSEP implemented several other strategies to homogenize and strengthen the position of EPs, like regionalization of the EM residency program, establishing postgraduate training courses, and the development of standards of care [2]. The goal of these measures was to create a common skill and knowledge base which will enable EPs to emancipate their position to one equal to other medical specialists.

Despite all these efforts, little research has been conducted regarding the current role and responsibilities of Dutch EPs, making it difficult to quantify their effect. One study described which organizational factors influence the position of EPs [3]; another showed an association between employment of EPs and the presence of postgraduate training and clinical audits in the ED [5]. However, both studies did not describe overall role or responsibilities of Dutch EPs. This gap in the literature led to two research questions which we aimed to answer:

1. What is the current position of EPs within hospital organizations?
2. Which roles and responsibilities do EPs have across Dutch EDs?

Results can be used to develop future strategies to further develop EM in the Netherlands. In addition, our findings might be useful to EM leaders in countries in an earlier stage of EM development.

## Methods

### Study setting

In the Netherlands, one hospital can incorporate more than one ED and emergency consultants working in these hospitals typically work in all ED locations [6]. Almost all EDs still work in a multidisciplinary model (EM is delivered by a collection of physicians of multiple specialties). This study uses the term “consultant body” (CB) to describe a group of emergency consultants working together for one hospital, possibly in multiple EDs. The term “main consultant” is used to describe the physician who carries medical and legal end-responsibility for the whole department or a subset of patients, while the “team leader” is the physician in charge of a specific team (e.g., CPR team). “Junior doctors” includes both junior doctors not involved in residency and residents.

At the time of inclusion, 85.1% (*n* = 74) of EDs were served by EPs through 56 consultant bodies (CBs). The EM residency program was taught in 39.2% (*n* = 29) of EP-staffed EDs. These programs were taught in both academic and non-academic hospitals. Academic hospitals are allied with Dutch universities (both in teaching medical students and in research) and deliver more tertiary care. The Netherlands count 8 academic hospitals of which 6 employ EPs. These 6 accounted for 8.1% of EP-staffed EDs and all offered EM residency programs. Non-academic hospitals accounted for 91.9% (*n* = 68) of EP-staffed EDs, and 33.8% (*n* = 23) of them offered EM residency programs [6–9].

### Study design

We performed a web-based cross-sectional survey among all EM consultant bodies in the Netherlands (CBs) in the first quarter of 2018 using SurveyMonkey. All EM CBs working in Dutch EDs were included, regardless of size. We did not have any exclusion criteria. For every CB, one EP was invited to answer on behalf of the CB. Respondents working in multiple locations could, for relevant questions, give separate answers for different locations.

### Measurements

The survey was designed by the researchers because no applicable survey existed. It aimed to capture the areas where we expected the highest variation in job profile, responsibilities, and autonomy. The questions posed can be broadly divided into three categories: baseline data, questions regarding the position within the organization, and questions regarding the role of the EP in the ED (see Additional file 1 for the complete survey).

### Data analysis

Descriptive statistics were used to describe the data. Analysis was performed using Statistical Package for the Social Sciences (SPSS) version 24. Questions were appropriately grouped per CB or ED.

## Results

The response rate was 91.1% of CBs, which included 87.7% of all EP-staffed EDs. However, there was some missing data (range 0.0–55.4%) in most questions. The 51 participating CBs consisted of 443 EPs. Together, these EPs covered 379.0 full-time equivalents (fte) of 36 h/week (see Table 1 for baseline data and Additional file 2 for the complete frequency tables).

**Table 1:** Baseline characteristics

	Frequency (%)	Median (IQR)	No. of missing answers (%)
Number of EPs	See Additional file 2	9 (6.0–11.25)	1 (2)
Number of female EPs	See Additional file 2	6 (4.0–7.0)	0 (0)
Number of male EPs	See Additional file 2	2 (2.0–4.0)	3 (5.9)
Number of fte	See Additional file 2	7.8 (5.17–9.7)	2 (4)
Number of years hospital has employed EPs	See Additional file 2	9.0 (7.0–11.0)	10 (19.6)
Average number of years of experience as EP for complete CB^a^			
0–5	14 (28.6)	N/A	2 (4)
5–10	30 (61.2)		
10–15	5 (10.2)		
Average age of EPs of CB in years^a^			
30–35	9 (18)	N/A	1 (2)
35–40	28 (56)		
40–45	13 (26)		
Contains EPs who completed emergency residency outside the Netherlands^b^			
Yes	2 (3.9)	N/A	0 (0)
Contains EBEEM-certified EPs^b^			
Yes	3 (5.9)	N/A	0 (0)
Number of ED locations			
1	39 (76.5)	1 (1.0–1.0)	0 (0)
2	10 (19.6)		
3	2 (3.9)		
Teaching hospital			
Yes	24 (47.0)	N/A	0 (0)
Academic hospital			
Yes	5 (9.8)	N/A	0 (0)

*EBEEM* European Board Examination in Emergency Medicine, *N/A* not applicable

^a^Asked as categorical variable, no median available

^b^All CBs for whom this was applicable replied that they employed one EBEEM-certified EP or EP trained abroad

### Position of EP in the hospital

The medical director of the department was an EP in 75.4% (*n* = 49) of EDs, while in 15.4% (*n* = 10), this was no EP. Missing data was 9.2% (*n* = 6). Most CBs were employed directly by the hospital (86.3%, *n* = 44), while 5.9% (*n* = 3) was employed through other medical specialists, and missing data was 7.8% (*n* = 4). Employment conditions were similar to those for other medical specialists on payroll for 37.3% (*n* = 19) of CBs, while 54.9% (*n* = 28) were hired under different conditions, and missing data was 7.8% (*n* = 4). A local document describing the responsibilities of EPs and other medical specialists regarding ED patients was present for 43.1% (*n* = 22) of CBs. This document was absent in 31.4% (*n* = 16), it was present but did not mention EPs in 3.9% (*n* = 2), and missing data was 21.6% (*n* = 11). EPs were the main consultant for all ED patients in 9.8% of CBs (*n* = 5), despite EM not having been recognized as a specialty on a national level. On the contrary, in 13.7% (*n* = 7) of CBs, EPs could not be the main consultant just because EM is not yet an official medical specialty. In several CBs (33.3%, *n* = 17), EPs could be the main consultants, but were limited to certain patient groups. These limits were related to referral status in 15.7% (*n* = 8) of CBs, to which resident (EM or different specialty) analyzes the patient in 3.9% (*n* = 2), to specialty in 2.0% (*n* = 1), or to disease in 2.0% (*n* = 1) and 11.8% (*n* = 6) remained unspecified. Missing data was 43.1% (*n* = 22).

Of the in-hospital staff federation, which represents the interests of medical specialists on board level, 88.2% (*n* = 45) of CBs were full members, 3.9% (*n* = 2) was not represented at all, and missing data was 7.8% (*n* = 4). There were several hospital-wide committees in which CBs were represented (see Table 2 for further specification). Presence of EPs in the ED 24/7 had been realized in 23.1% (*n* = 15) of EDs; 4.6% of EDs (*n* = 3) had their previous 24/7 presence disrupted due to personnel problems. Coverage was highest during day shift on weekdays, when in 72.3% of EDs (*n* = 47), an EP was always present; in 3.1% of EDs (*n* = 2), an EP was sometimes present; and missing data was 24.6% (*n* = 16). Coverage was lowest during night shifts on weekdays, when in 23.1% of EDs (*n* = 15), an EP was always present, sometimes present in 6.2% (*n* = 4), never present in 46.2% of EDs (*n* = 30), and missing data was 24.6% (*n* = 16) (see Additional file 3 for specification per shift). In case of overcrowding, 58.8% (*n* = 30) of CBs had the authority to instruct emergency medical services (EMS) to deviate to other hospitals. 5.9% of CBs (*n* = 3) did not have this authority, and 11.8% of CBs (*n* = 6) had the authority to suggest this measure, but it was executed by others. Missing data was 13.8% (*n* = 7), and rerouting of EMS did not occur in 9.8% of CBs (*n* = 5), for example, in remote areas.

**Table 2:** Participation in hospital committees or boards

Committee or board name	Participating, *N* (%)^a^	Not participating, *N* (%)^a^	Subtotal, *N*	Not present, *N* (%)^b^	No data, *N* (%)^b^
CPR	43 (91.5)	4 (8.5)	47	0 (0.0)	4 (7.8)
PSA	42 (100.0)	0 (0.0)	42	5 (9.8)	9 (17.6)
Child abuse	45 (97.8)	1 (2.2)	46	1 (2.0)	4 (7.8)
Elder abuse	35 (92.1)	3 (7.9)	38	9 (17.6)	4 (7.8)
Organ donation	26 (65.0)	14 (35.0)	40	6 (11.8)	5 (9.8)
Antibiotics	8 (20.5)	31 (79.5)	39	5 (9.8)	7 (13.7)
Medication	18 (51.4)	17 (48.6)	35	9 (17.6)	7 (13.7)
Quality and safety	30 (71.4)	12 (28.6)	42	4 (7.8)	5 (9.8)
Science	13 (39.4)	20 (60.6)	33	12 (23.5)	6 (11.8)
Appointment advisory	23 (59.0)	16 (41.0)	39	7 (13.7)	12 (23.5)
Investment	4 (12.1)	29 (87.9)	33	10 (19.6)	8 (15.7)

*CPR* cardiopulmonary resuscitation, *PSA* procedural sedation and analgesia

^a^The number of hospitals in which the committee is the denominator of these percentages

^b^The total number of CBs is the denominator of these percentages

### Role of EP in the ED

Respondents were asked about the local division of workload based on referral status. Self-referred patients were analyzed by EPs in 75.4% of EDs (*n* = 49), and missing data was 24.6% (*n* = 16). Referred patients were analyzed by EPs in 73.9% (*n* = 48) of EDs, EPs do not analyze referred patients in one ED (1.5%), and missing data was similar at 24.6% (*n* = 16). Within the referred patients subgroup, EPs analyzed patients for all specialties in 26.2% (*n* = 17) of EDs; however, specialty-bound limits for EPs to analyze these patients were present in 47.7% of EDs (*n* = 31). Missing data was 29.2% (*n* = 19) (see Additional file 4 for specification per specialty). Other limits mentioned included current work pressure for the different specialties in 15.4% of EDs (*n* = 10), time of day in 3.1% (*n* = 2), and which specific illness was suspected in 3.1% (*n* = 2) of EDs.

A separate *First Heart Aid* (“Eerste Hart Hulp,” EHH), in which patients with presumed cardiac pathology are analyzed, was present in 49.2% of EDs (*n* = 32). Patients were treated solely by cardiology in 75.0% of these units (*n* = 24), while 25.0% (*n* = 8) were run as a cooperation between cardiologists and EPs. There was no EHH in 36.9% of EDs (*n* = 24), and missing data was 13.8% (*n* = 9).

Which procedures were performed by all consultants of the CB is detailed in Table 3, and which roles EPs have when working in specific teams is detailed in Table 4. It is of interest to note that team leadership and end-responsibility did not always go hand in hand; in fact, EPs were more often team leaders than that they carry official end-responsibility.

**Table 3:** Procedures performed by all consultants in consultant body

Procedure	Performed, *N* (%)	Not performed by all EPs or data missing, *N* (%)
PSA in adults	40 (78.4)	11 (21.6)
PSA in children > 12	28 (54.9)	23 (45.1)
PSA in children 8–12	24 (47.1)	27 (52.9)
PSA in children 4–12	21 (41.2)	30 (58.8)
PSA in all children	5 (9.8)	46 (90.2)
Emergency intubation	13 (25.5)	38 (74.5)
Electrocardioversion	18 (35.3)	33 (64.7)
Lumbar puncture	3 (5.9)	48 (94.1)
Chest drains	39 (76.5)	12 (23.5)
Fascia iliac compartment block	11 (21.6)	40 (78.4)
EFAST	26 (51.0)	25 (49.0)
Cardiac US	13 (25.5)	38 (74.5)
Aortic US	20 (39.2)	31 (60.8)
IVC US	14 (27.5)	37 (72.5)
Lung US	14 (27.5)	37 (72.5)
Complete emergency US^a^	11 (21.6)	40 (78.4)

*PSA* procedural sedation and analgesia, *EFAST* extended focused assessment with sonography for trauma, *US* ultrasound, *IVC* inferior vena cava

^a^Complete emergency US is a package which contains the five domains mentioned above. The DSEP is training all EPs in this package

**Table 4:** Roles within teams

Team name	Main consultant (%)	Team leader (%)	Treatment A/B (%)	Other standard role (%)	No standard role (%)	Team not present (%)	No data (%)
CPR in ED	33 (50.8)	48 (73.8)	14 (21.5)	5 (7.7)	4 (6.2)	0 (0.0)	9 (13.8)
CPR in hospital	14 (21.5)	24 (36.9)	11 (16.9)	2 (3.1)	25 (38.5)	0 (0.0)	11 (16.9)
Large trauma	20 (30.8)	33 (50.8)	6 (9.2)	10 (15.4)	2 (3.1)	13 (20.0)	10 (15.4)
Small trauma	31 (47.7)	41 (63.1)	10 (15.4)	6 (9.2)	2 (3.1)	11 (16.9)	9 (13.8)
Stroke	16 (24.6)	33 (50.8)	11 (16.9)	7 (10.8)	7 (10.8)	5 (7.7)	10 (15.4)
RAAA	19 (29.2)	37 (56.9)	8 (12.3)	5 (7.7)	3 (4.6)	11 (16.9)	9 (13.8)

Selection of multiple roles per team was possible. Percentages are calculated with the total number of EDs as denominator

*CPR* cardiopulmonary resuscitation, *RAAA* ruptured abdominal aortic aneurysm, *A/B* airway/breathing

EPs supervise EM junior doctors in 78.5% of EDs (*n* = 51), general practitioners (GPs) in training in 80.0% (*n* = 52), and junior doctors of other specialties in 41.5% (*n* = 27). Missing data was 21.5%, 20.0%, and 58.5%, respectively. CBs also mentioned supervising medical interns, military doctors in training, physician assistants, and nurse practitioners.

In-hospital tasks for EPs included care on the wards outside office hours for 10.7% (*n* = 7) of EDs; this was 9.2% (*n* = 6) for the intensive care unit (ICU). In-hospital tasks remained unspecified for 7.7% of EDs (*n* = 5), there were no in-hospital tasks for 15.4% (*n* = 10), and missing data was 55.4% (*n* = 36). EPs are part of the medical emergency team (MET) in 21.5% (*n* = 14) of EDs.

## Discussion

The perceived heterogeneity that caused us to conduct this study is clearly demonstrated by several of our results, of which we will highlight the main ones here. An important example is the difference in physical presence of EPs. We found 24/7 presence of EPs in 23.1% of EDs, which is quite an improvement compared to the 6% measured in 2011, but is still a long way off from the desired 100% [1]. At least five CBs report vacancies for EPs, while paradoxically, the number of residents has decreased. Because the EP workforce is relatively young and is foreseen to remain part of the active workforce for years to come, several stakeholders, including the DSEP, advocated for this decrease to prevent unemployment in the long term. The workforce is expected to have grown sufficiently to cover all vacancies in about 10 years’ time [10]. Future presence of EPs 24/7 in every ED is one of the main viewpoints of the DSEP [2, 11]. Presence of EPs 24/7 in all EDs ensures continuity of delivery of emergency care and is essential for improvement thereof. Legislation about which physician can be main consultant is interpreted differently by individual hospitals. This is illustrated by two of our findings. Firstly, that although all hospitals work under the same legislation, for 9.8% of CBs, EPs are allocated this role by default for all ED patients, while in 13.7% of CBs, EPs cannot perform this role at all. Secondly, EPs are more often team leader than that they are main consultant. To our knowledge, no measures have been undertaken to address this discrepancy. EPs being main consultant for all ED patients might increase adherence to standards of emergency care.

Another factor showing heterogeneity is the difference in employment conditions between CBs. Despite working in a stressful environment with a high workload [12], less than half of CBs (37.3%) are awarded in accordance with the medical specialists’ collective agreement. We hypothesize that the status of EM as an area of special interest instead of a medical specialty is largely the cause of this discrepancy.

Variety in the role of the EP in the ED is clearly visible in disparities in which procedures are performed by all members of CBs, with a substantial range between the different procedures (5.9–78.4%). Heterogeneous training of EPs, variation in task allocation (e.g., in most hospitals neurologists perform all LPs in the ED, but in some hospitals this task is allocated to EPs), and differences in standards of care (e.g., not all EDs offer PSA) all contribute to this heterogeneity. A common foundation in the skillset of EPs and professional standardization are prerequisites to further professionalize emergency medicine in the Netherlands.

The presence or absence of an *EHH* run solely by cardiologists is another cause of heterogeneity. It is of concern to see that in 36.9% of EDs, EPs have no role in analyzing and treating patients in the *EHH*, which hampers exposure to cardiac pathology. This creates disparities between EPs within the Netherlands as well as when comparing Dutch EPs to their international counterparts, for whom the management of acute cardiac disease is everyday business. No specific strategies have been employed to ensure adequate exposure to cardiac pathology for all EPs, but the issue is addressed to some extent by the DSEP postgraduate training programs which include cardiac care.

The last cause of heterogeneity we would like to mention is supervision of junior doctors. Although supervision of EM junior doctors and GPs in training is a common task, this clearly contrasts with the infrequent supervision of junior doctors of other specialties. This has two potential negative effects. Firstly, it could lead to fragmented care in the ED, with patients receiving different care depending on which resident analyzes them. Secondly, differences in supervision make it more difficult to establish a uniform EP role model for future EPs and upcoming medical specialists.

Previous research has shown that development of the EP role is influenced by institutional policies and the role EPs allocate themselves as well as legislation, training programs, support of medical managers, and hospital culture [3]. Our study highlights heterogeneity in task allocation and institutional policies as possible inhibiting factors of EP emancipation. It is our opinion that in order to improve emergency medicine in the Netherlands, it is necessary to reduce this heterogeneity. Measures taken should include attention for postgraduate training, ongoing development of standards of care, more uniformity in residency programs, extension of the current 3-year curriculum to the European minimum of 5 years and recognition of EM as an independent specialty [13]. International comparison of our data unfortunately proved impossible. Firstly, we could not find any comparable literature. One previous paper compared the development of EM between the UK and the Netherlands, focusing on the historic background, cultural mandate, and institutional license. It does not quantify tasks or policies [14]. Secondly, the argument has been made that international comparison is impossible due to differences in health care systems [15].

Strengths of the study include the very high response rate and the novelty of this data, as there are no previous studies in this specific area. Limitations include the response rate per question, which was considerably lower than the overall response rate. This was possible because most questions were non-obligatory, which was a deliberate policy; we worked under the assumption that we would gain more generalizable data due to a higher overall response rate.

A second limitation is that there was no validated questionnaire available and we have not validated our self-designed survey. Developing our own allowed us to inquest our specific interest, but might have left room for misinterpretation. It would have been of great interest to study perceived barriers in advancing EM using open questions, but that was beyond the scope of this study.

## Conclusion

We conclude that there is a high degree of heterogeneity between emergency consultant bodies across the Netherlands, both in regard to position in the hospital and responsibilities in the ED. Lack of uniformity might inhibit emancipation of the emergency medicine discipline. The way forward is to create more homogeneity by training both EPs and residents in a more uniform manner, developing standards of care, and recognition of EM as an independent specialty.

## Supplementary information

**Additional file 1.** Complete survey (Dutch).

**Additional file 2.** Baseline data frequency tables.

**Additional file 3.** EP presence per shift.

**Additional file 4.** Referred patients analyzed by EPs per specialty.

## Data Availability

The datasets generated and analyzed during the current study are not publicly available due to privacy reasons, but are available from the corresponding author on reasonable request.

## Abbreviations

A/B: Airway/breathing
CB(s): Consultant body(ies)
CPR: Cardiopulmonary resuscitation
DSEP: Dutch Society of Emergency Physicians
EBEEM: European Board Examination in Emergency Medicine
ED(s): Emergency department(s)
EFAST: Extended focused assessment with sonography for trauma
EHH: First Heart Aid/*Eerste Hart Hulp*
EM: Emergency medicine
EMS: Emergency medical services
EP(s): Emergency physician(s)
Fte: Full-time equivalents
GP: General practitioner
ICU: Intensive care unit
IVC: Inferior vena cava
LP: Lumbar puncture
MET: Medical emergency team
N/A: Not applicable
PSA: Procedural sedation and analgesia
RAAA: Ruptured abdominal aortic aneurysm
SPSS: Statistical Package for the Social Sciences
US: Ultrasound
