# Supplementary material for: Position within the hospital and role in the emergency department of emergency physicians in the Netherlands: a national survey
Source: Int J Emerg Med. 2020 Feb 10;13:8. doi: 10.1186/s12245-020-0267-2 (PMC7011557; doi:10.1186/s12245-020-0267-2)
Supplement: Supplementary file 1 — Additional file 1. Complete survey (Dutch). [file 12245_2020_267_MOESM1_ESM.docx]

Additional File 1 – complete survey (Dutch)

Legenda:

● optie van multiple choice vraag, één antwoord mogelijk

€ optie van multiple choice vraag, meerdere antwoorden mogelijk

* deze vragen werden per locatie gesteld, wat betekent dat zij werden herhaald bij vakgroepen die aangaven op meerdere locaties te werken.

Deel 1: algemene vragen

1. Voor welk(e) ziekenhuis/ziekenhuizen is de vakgroep werkzaam? Graag zowel de naam als de plaats noemen.
2. Op hoeveel locaties is de vakgroep werkzaam?
   - 1
   - 2
   - 3
3. Sinds wanneer is de eerste SEH-arts werkzaam in uw ziekenhuis (als SEH-arts^knmg^)?
4. Uit hoeveel personen bestaat de vakgroep in januari 2018?

Aantal personen:

Aantal vrouwen:

Aantal mannen:

1. Uit hoeveel FTE (waarbij 1 fte = 36 uur) bestaat de vakgroep in januari 2018?
2. Wat is de gemiddelde leeftijd van de leden van de vakgroep?
   - 25 – 30 jaar
   - 30 – 35 jaar
   - 35 – 40 jaar
   - 40 – 45 jaar
3. Hoeveel jaar zijn de leden van de vakgroep, gemiddeld genomen, RGS-geregistreerd als SEH-arts^knmg^?
   - 0 – 5 jaar
   - 5 – 10 jaar
   - 10 – 15 jaar
4. Zijn er leden van de vakgroep die hun volledige opleiding tot SEH-arts/Emergency Physician (EP) in het buitenland hebben gevolgd?
   - Nee
   - Ja. Vul in hoeveel leden in het buitenland zijn opgeleid en waar deze zijn opgeleid.
5. Zijn er leden van de vakgroep die EBEEM (EUSEM)-gecertificeerd zijn?
   - Nee
   - Ja. Vul in hoeveel leden EBEEM-gecertificeerd zijn en waar zij dit certificaat hebben behaald.

Deel 2: organisatorische positionering in het ziekenhuis

1. Vormen de SEH-artsen^knmg^ een unieke en zelfstandige vakgroep binnen het ziekenhuis?
   - Ja
   - Nee
2. Is er altijd, 24/7, een SEH-arts^knmg^ fysiek op de SEH aanwezig?*
   - Ja
   - Nee. Beschrijf op welke uren/dagen de SHE-arts fysiek aanwezig is en, indien van toepassing, op welke momenten er sprake is van supervisie op afstand.
3. Is het medisch hoofd/de medisch manager van de SEH een SEH-arts^knmg^?*
   - Ja
   - Nee
4. Bij wie zijn de SEH-artsen^knmg^ in loondienst?
   - Ziekenhuis
   - Medisch Specialistisch Bedrijf/Coöperatie Vrijgevestigden
   - Anders, namelijk:
5. Onder welke arbeidsvoorwaardenregeling vallen de SEH-artsen^knmg^?
   - CAO Ziekenhuizen functiegroep 75
   - CAO Ziekenhuizen functiegroep 80
   - CAO Universitair Medische Centra
   - Arbeidsvoorwaardenregeling Medisch Specialisten (AMS/UMS). Vul bij “andere bepalingen”alstublieft de hoogte van de inconveniëntentoeslag in.
   - Andere relevante bepalingen of regelingen, of vul hier de inconveniëntentoeslag in:
6. Op welke manier zijn SEH-artsen^knmg^ onderdeel van de Vereniging of Federatie Medisch Specialisten (VMS/FMS) binnen het ziekenhuis?
   - Volwaardig lid
   - Buitengewoon lid
   - Niet vertegenwoordigd
   - Anders, namelijk:
7. Hebben SEH-artsen^knmg^ zitting in:
   - Het bestuur van de VMS/FMS
   - Het bestuur van de Vereniging Medisch Specialisten in Dienstverband
8. Hebben SEH-artsen^knmg^ zitting in ziekenhuisbrede commissies/werkgroepen?

|  | Nemen WEL zitting | Nemen GEEN zitting | Commissie/werkgroep niet aanwezig |
| --- | --- | --- | --- |
| Reanimatiecommissie | ● | ● | ● |
| PSA-commissie | ● | ● | ● |
| Werkgroep kindermishandeling | ● | ● | ● |
| Werkgroep ouderenmishandeling | ● | ● | ● |
| Donatie werkgroep | ● | ● | ● |
| Antibiotica werkgroep | ● | ● | ● |
| Medicatie werkgroep | ● | ● | ● |
| Kwaliteit & veiligheid | ● | ● | ● |
| Wetenschapscommissie | ● | ● | ● |
| Benoemingsadviescommissie (BAC) | ● | ● | ● |
| Investeringscommissie | ● | ● | ● |
| Andere commisies/werkgroepen, namelijk: | | | |

1. Wat staat in het ‘ziekenhuisbrede verantwoordelijkheidsdocument’ t.a.v. de SEH-arts^knmg^ beschreven? Graag passage(s) woordelijk delen.
2. Indien separaat aanwezig: wat staat in het ‘document hoofdbehandelaarschap’ t.a.v. de SEH-arts^knmg^ beschreven? Graag passage(s) woordelijk delen.

Deel 3: eindverantwoordelijkheid op de SEH

Deel 3A: organisatorische eindverantwoordelijkheid

1. Welke patiënten worden door (of onder supervisie van) de SEH-arts^knmg^  gezien en behandeld?*
   - Zelfverwijzers
   - Presentatie van patiënten per 112 melding (“free calls”)
   - Verwezen patiënten voor de volgende specialismen:
2. Is overleg met een medisch specialist noodzakelijk als de SEH-arts^knmg^ patiënten vanaf de SEH naar huis (dus zonder follow-up in het ziekenhuis) ontslaat?*
   - Ja, voor alle patiënten die naar huis worden ontslagen is overleg noodzakelijk
   - Nee, voor geen enkele patiënt die naar huis wordt ontslagen is overleg noodzakelijk
   - Nee, voor een deel van de patiënten/specialismen is overleg niet noodzakelijk. Vul in welke patiënten u zonder overleg naar huis ontslaat:
3. Is overleg met een medisch specialist noodzakelijk als de SEH-arts^knmg^ patiënten vanaf de SEH ontslaat en ze terug laat komen op de polikliniek?*
   - Ja, voor alle patiënten die poliklinisch retour komen is overleg noodzakelijk
   - Nee, voor geen enkele patiënt die poliklinisch retour komt is overleg noodzakelijk
   - Nee, voor een deel van de patiënten/specialismen is overleg niet noodzakelijk. Vul in welke patiënten u zonder overleg poliklinisch terug laat komen:
4. Is overleg met een medisch specialist noodzakelijk als de SEH-arts^knmg^ patiënten vanaf de SEH opneemt?*
   - Ja, voor alle patiënten die opgenomen worden is overleg noodzakelijk
   - Nee, voor geen enkele patiënt die opgenomen wordt is overleg noodzakelijk
   - Nee, voor een deel van de patiënten/specialismen is overleg niet noodzakelijk. Vul in welke patiënten u zonder overleg opneemt:
5. Heeft de SEH-arts^knmg^ mandaat om in geval van crowding een ‘ambulance stop’ af te kondigen?
   - Ja
   - Nee
   - Anders, namelijk:
6. Heeft de SEH-arts^knmg^ mandaat om in geval van crowding of een calamiteit te interveniëren in zorgprocessen (escaleren of opschalen)?
   - Ja
   - Nee
   - Anders, namelijk:
7. Heeft de SEH-arts^knmg^ de eindverantwoordelijkheid/het hoofdbehandelaarschap over één of meerdere van de volgende multidisciplinaire teams?*
   - Reanimatie op SEH
   - Reanimatie buiten SEH
   - Groot traumateam
   - Klein traumateam
   - Opvang CVA
   - Opvang AAAA
   - Ander(e) multidisciplinair(e) team(s), namelijk:

Deel 3B: medisch inhoudelijke eindverantwoordelijkheid

1. Welke artsen op de SEH worden door de SEH-arts^knmg^ gesuperviseerd?*
   - A(N)IOS SEG
   - A(N)IOS andere specialismen
   - Huisartsen in opleiding
   - Anders, namelijk:
2. Is er in het ziekenhuis een separate EHH/EHL/CCU (dus niet onder de verantwoordelijkheid van de vakgroep SEG) waar ongedifferentieerde patiënten met bijvoorbeeld thoracale pijn of palpitaties worden gezien?*
   - Ja
   - Nee
   - Anders, namelijk:
3. Welke rol heeft de SEH-arts^knmg^ in de volgende multidisciplinaire teams?*

|  | Coördinatie van de opvang | Behandeling A/B | Andere vaste rol | Geen vaste rol/niet betrokken bij de opvang | Niet aanwezig in het ziekenhuis |
| --- | --- | --- | --- | --- | --- |
| Reanimatie op SEH | € | € | € | € | € |
| Reanimatie buiten SEH | € | € | € | € | € |
| Groot traumateam | € | € | € | € | € |
| Klein traumateam | € | € | € | € | € |
| Opvang CVA | € | € | € | € | € |
| Opvang AAAA | € | € | € | € | € |
| Rol in een ander team, namelijk: | | | | | |

1. Welke van de volgende verrichtingen worden door alle SEH-artsen^knmg^ van uw vakgroep zelfstandig uitgevoerd?
   - Procedurele sedatie en analgesie (PSA) bij volwassenen
   - PSA bij kinderen ≥ 12 jaar oud
   - PSA bij kinderen 8 – 12 jaar oud
   - PSA bij kinderen 4 – 12 jaar oud
   - PSA bij alle kinderen ongeacht leeftijd
   - Acuut luchtwegmanagement inclusief spoedintubaties
   - Elektrocardioversie niet gerelateerd aan reanimatie
   - Lumbaalpuncties
   - Plaatsen thoraxdrain
   - Extended Focused Assessment Sonography in Trauma (EFAST)
   - Echocardiografie
   - Echografie abdominale aorta
   - Echografie vena cava inferior
   - Longechografie
   - Fascia Iliaca Compartiment Block (FICB)
   - Anders, namelijk:
2. Verricht de SEH-arts^knmg^ ook structurele werkzaamheden op andere afdelingen (dus méér dan bijvoorbeeld een reanimatie)?*
   - In de dienst voorwacht voor de zalen
   - In de dienst “tussenwacht” voor de zalen: de A(N)IOS SEG heeft voorwacht en overlegd éérst met de SEH-arts voordat de specialist betrokken raakt
   - In de dienst voorwacht voor de Intensive Care
   - Tijdens kantooruren werkzaamheden op de Intensive Care of zalen
   - Onderdeel Spoed Interventie Team (SIT)
   - Anders, namelijk:
3. Opmerkingen of vragen:
