# Supplementary material for: Position within the hospital and role in the emergency department of emergency physicians in the Netherlands: a national survey
Source: Int J Emerg Med. 2020 Feb 10;13:8. doi: 10.1186/s12245-020-0267-2 (PMC7011557; doi:10.1186/s12245-020-0267-2)
Supplement: Supplementary file 2 — Additional file 2. Baseline data frequency tables. [file 12245_2020_267_MOESM2_ESM.docx]

**Additional file 2 – baseline data frequency tables**

| **Table 1: how many EPs does the CB consist of?** | | |
| --- | --- | --- |
| Number of persons | Frequency | Percent |
| 2 | 1 | 2.0 |
| 3 | 3 | 5.9 |
| 4 | 2 | 3.9 |
| 5 | 4 | 7.8 |
| 6 | 4 | 7.8 |
| 7 | 4 | 7.8 |
| 8 | 5 | 9.8 |
| 9 | 5 | 9.8 |
| 10 | 6 | 11.8 |
| 11 | 4 | 7.8 |
| 12 | 6 | 11.8 |
| 13 | 3 | 5.9 |
| 15 | 1 | 2.0 |
| 17 | 1 | 2.0 |
| 20 | 1 | 2.0 |
| Subtotal | 50 | 98.0 |
| Missing | 1 | 2.0 |
| Total | 51 | 100.0 |

| **Table 2: how many of the EPs in the CB are women?** | | |
| --- | --- | --- |
| Number of persons | Frequency | Percent |
| 1 | 2 | 3.9 |
| 2 | 1 | 2.0 |
| 3 | 8 | 15.7 |
| 4 | 5 | 9.8 |
| 5 | 7 | 13.7 |
| 6 | 8 | 15.7 |
| 7 | 8 | 15.7 |
| 8 | 5 | 9.8 |
| 9 | 2 | 3.9 |
| 10 | 3 | 5.9 |
| 13 | 2 | 3.9 |
| Total | 51 | 100.0 |

| **Table 3: how many of the EPs in the CB are men?** | | |
| --- | --- | --- |
| Number of persons | Frequency | Percent |
| 0 | 2 | 3.9 |
| 1 | 7 | 13.7 |
| 2 | 16 | 31.4 |
| 3 | 7 | 13.7 |
| 4 | 6 | 11.8 |
| 5 | 3 | 5.9 |
| 6 | 3 | 5.9 |
| 7 | 4 | 7.8 |
| Subtotal | 48 | 94.1 |
| Missing | 3 | 5.9 |
| Total | 51 | 100.0 |

| **Table 4: of how many fte does the CB consist?^a^** | | |
| --- | --- | --- |
| Categories | Frequency | Percent |
| 0-5 fte | 12 | 23.5 |
| 5-10 fte | 28 | 54.9 |
| 10-15 fte | 8 | 15.7 |
| >15 fte | 1 | 2.0 |
| Subtotal | 49 | 96.1 |
| Missing | 2 | 3.9 |
| Total | 51 | 100.0 |

^a^ the question was asked as a numerical value. The frequency table for this question consisted of many different values, e.g. 7.0, 7.2 etc, all with frequency 1. Thus we chose the represent the data in the categories displayed here.

| **Table 5: how many years has the hospital employed EPs?** | | |
| --- | --- | --- |
| Number of years | Frequency | Percent |
| 0 | 1 | 2.0 |
| 3 | 1 | 2.0 |
| 4 | 2 | 3.9 |
| 5 | 2 | 3.9 |
| 6 | 3 | 5.9 |
| 7 | 5 | 9.8 |
| 8 | 4 | 7.8 |
| 9 | 8 | 15.7 |
| 10 | 2 | 3.9 |
| 11 | 5 | 9.8 |
| 12 | 3 | 5.9 |
| 13 | 2 | 3.9 |
| 14 | 2 | 3.9 |
| 28 | 1 | 2.0 |
| Subtotal | 41 | 80.4 |
| Missing | 10 | 19.6 |
| Total | 51 | 100.0 |

| **Table 5b: how many years has the hospital employed EPs? In categories^a^** | | | |
| --- | --- | --- | --- |
| Category | Frequency | | Percent |
| 0-5 years | 6 | 11.8 | |
| 6-10 years | 22 | 43.1 | |
| 11-15 years | 12 | 23.5 | |
| >15 years | 1 | 2.0 | |

^a^ the question was asked as a numerical value, categories were added by the researchers.
