# Supplementary material for: Position within the hospital and role in the emergency department of emergency physicians in the Netherlands: a national survey
Source: Int J Emerg Med. 2020 Feb 10;13:8. doi: 10.1186/s12245-020-0267-2 (PMC7011557; doi:10.1186/s12245-020-0267-2)
Supplement: Supplementary file 3 — Additional file 3. EP presence per shift. [file 12245_2020_267_MOESM3_ESM.docx]

**Additional file 3 – EP presence per shift**

**Day shift is defined as presence in the ED between 8:00-16:00, evening shift as 16:00-24:00, and night shift as 24:00-8:00. However, we were not strict in this definition because of the variable start & end times of shifts. We counted shifts which were maximal 2 hours shorter as defined as a complete shift; if more than 2 hours, we counted half a shift. For example, a 10:00-19:00 shift counts as a complete day shift but half an evening shift. Missing data was 24.6% (n=16), and these responses were excluded in the table and figure. The total number of EDs included is 65.**

| Shift | Number of EDs in which an EP is present | | | |
| --- | --- | --- | --- | --- |
|  | Always | Sometimes | Never | Half shift |
| Day shift, weekdays | 47 | 2 | 0 | 0 |
| Evening shift, weekdays | 37 | 6 | 5 | 1 |
| Night shift, weekdays | 15 | 4 | 30 | 0 |
| Day shift, weekends | 44 | 2 | 2 | 1 |
| Evening shift, weekends | 32 | 6 | 9 | 2 |
| Night shift, weekends | 18 | 3 | 28 | 0 |

Table 1: number of EDs in which an EP is present during a specific type of shift

Figure 1: number of EDs in which an ED is present during a specific type of shift
