# Supplementary material for: Position within the hospital and role in the emergency department of emergency physicians in the Netherlands: a national survey
Source: Int J Emerg Med. 2020 Feb 10;13:8. doi: 10.1186/s12245-020-0267-2 (PMC7011557; doi:10.1186/s12245-020-0267-2)
Supplement: Supplementary file 4 — Additional file 4. Referred patients analyzed by EPs per specialty. [file 12245_2020_267_MOESM4_ESM.docx]

**Additional file 4 – Referred patients analyzed by EPs per specialty**

**Missing data was 24.6% (n=16) of EDs, and 4.6% (n=3) gave unclear answers. These respondents are excluded from both the table and figure. The total number of EDs included is 65.**

|  | Referred patients can be analyzed by EPs | | |
| --- | --- | --- | --- |
| Specialty | Never | Always | Sometimes |
| Cardiology | 10 | 32 | 4 |
| Gastroenterology | 8 | 36 | 2 |
| Geriatrics | 12 | 31 | 3 |
| Internal medicine | 5 | 35 | 6 |
| Neurology | 7 | 36 | 3 |
| O&G | 21 | 23 | 2 |
| OMS | 8 | 36 | 2 |
| Opthalmology | 11 | 33 | 2 |
| Orthopedics | 5 | 39 | 2 |
| Otolaryngology | 3 | 41 | 2 |
| Pediatrics | 20 | 22 | 4 |
| Plastic surgery | 7 | 37 | 2 |
| Pulmonology | 3 | 41 | 2 |
| Surgery | 1 | 43 | 2 |
| Urology | 6 | 38 | 2 |

**Table 1: number of EDs in which local agreements allow EPs to analyze referred patients, sorted by specialty.**

**Abbreviations used: O&G ‘Obstetrics & Gynecology’, OMS ‘Oral and Maxillofacial Surgery’.**

**Figure 1: number of EDs in which local agreements allow EPs to analyze referred patients, sorted by specialty.**

**Abbreviations used: O&G ‘Obstetrics & Gynecology’, OMS ‘Oral and Maxillofacial Surgery’.**
